# Comparative analysis of immune escape and BCR repertoire remodeling after Omicron BF.7 and JN.1 infections

**DOI:** 10.3389/fimmu.2026.1906350

**Published:** 2026-08-25

**Authors:** Luanfeng Lin, Luxuan Yang, Yulei Sun, Jiayan Li, Yinying Lu, Hongyu Wang, Yabo Mi, Liangjiu Zhang, Binhuang Sun, Xinyi Yu, Tianmei Yu, Xiaoyu Zhao, Jingwen Ai, Wenhong Zhang

**Affiliations:** 1 The First Clinical College of Fujian Medical University, the First Affiliated Hospital of Fujian Medical University, Fuzhou, China; 2 Department of Infectious Disease, Mengchao Hepatobiliary Hospital of Fujian Medical University, Fuzhou, China; 3 Shanghai Sci-Tech Inno Center for Infection & Immunity, National Medical Center for Infectious Diseases, Huashan Hospital, Institute of Infection and Health, Fudan University, Shanghai, China; 4 Department of Infectious Diseases, Shanghai Key Laboratory of Infectious Diseases and Biosafety Emergency Response, National Medical Center for Infectious Diseases, Huashan Hospital, Fudan University, Shanghai, China; 5 Department of Infection Medicine Center, National Regional Medical Center, Binhai Campus of the First Affiliated Hospital, Fujian Medical University, Fuzhou, China

**Keywords:** antigenic cartography, BCR repertoire, neutralization breadth, Omicron, SARS-CoV-2

## Abstract

SARS-CoV-2 Omicron evolution continues to erode antibody-mediated immunity, but whether infection with antigenically distinct lineages simply amplifies pre-existing memory or redirects humoral recognition toward newly evolved antigenic space remains unclear. Here, we analyzed paired early- and convalescent-phase plasma and whole-blood samples from individuals with sequencing-confirmed BF.7 or JN.1 infection. Integrated neutralization profiling, antigenic cartography, and bulk B cell receptor (BCR) repertoire analysis revealed lineage-associated humoral trajectories. Both breakthrough infections increased cross-variant neutralization, but their updated humoral profiles were not equivalent. BF.7 breakthrough infection broadly boosted neutralization from a BA.5-related background, whereas JN.1 breakthrough infection produced greater gains against BA.2.86, JN.1, and KP.2 and shifted humoral recognition toward late-Omicron antigenic space. Neutralization remained branch-structured, with BQ.1.1 and KP.2 acting as persistent escape nodes within the BA.2/BA.5-related and BA.2.86/JN.1-related branches, respectively. This humoral updating paralleled changes in IGH clonotype counts, repertoire diversity, V/J gene usage, isotype composition, and V–J pairing, whereas major CDR3 length distributions and sequence-logo patterns remained largely conserved. Overall, these findings indicate that antigenically distinct Omicron breakthrough infections generate non-equivalent humoral recall trajectories: BF.7 exposure preferentially reinforced BA.5-related antibody recognition, whereas JN.1 exposure more effectively redirected recognition toward BA.2.86/JN.1-derived variants.

## Introduction

Since the emergence of Omicron, SARS-CoV-2 has continued to diversify through stepwise accumulation of spike mutations that reshape antigenic relationships and neutralization sensitivity. BF.7 and JN.1 represent two antigenically distinct stages of this process: BF.7 belongs to a BA.5-related background, whereas JN.1 arose from the BA.2.86-derived lineage and subsequently became a major late-Omicron variant (1, 2). Although national surveillance in China indicated that JN.1 was predominant by mid-2024, regional sequencing in Fujian detected both BF.7 and JN.1 during the 2024 transition period, providing a natural setting to compare humoral responses after infection by divergent Omicron sublineages (3). This setting allowed the antigenic effects of infecting variants to be assessed within a shared regional context rather than inferred solely from chronological lineage replacement.

In populations shaped by vaccination and repeated infection, Omicron exposure rarely occurs on an immunologically naive background. Unlike primary infection, exposure after prior antigenic experience can recall pre-existing cross-reactive memory B cells, expand established clonotypes, and selectively remodel antibody specificity rather than generate a purely *de novo* response (4). Repeated Omicron exposures can partially overcome ancestral immune imprinting, indicating that immune recall is plastic and can be reshaped by the antigenic character and spacing of subsequent exposures (5). This concept is supported by our previous work showing that successive Omicron sublineages differ in antibody escape and that booster vaccination or breakthrough infection can restore neutralization breadth in a variant-dependent manner (6, 7). Subsequent analyses of BA.2.86/JN.1 and XBB-era variants we further revealed lineage-specific immune evasion, altered virological properties, and antigenic separation among divergent branches (8). Together, these observations suggest that the outcome of Omicron infection is jointly shaped by host immune history and the antigenic position of the infecting variant.

However, whether distinct late-Omicron lineages leave separable humoral imprints remains incompletely defined. Studies of BA.1- and XBB-era immune exposure have shown that antigenically shifted Omicron variants can broaden neutralization and recall pre-existing memory B-cell responses, while antigenic cartography has shown that bivalent vaccine antigen choice can reshape neutralization landscapes (9–11). Together with our recent work showing progressive antibody escape from early Omicron sublineages and BA.5-related variants to antigenically divergent lineages such as EG.5.1/HK.3 and BA.2.86/JN.1, these findings establish the variant-dependent nature of Omicron immune escape (6–8). They do not, however, determine whether antigenically divergent infections remodel humoral immunity along distinct trajectories. In particular, BF.7, representing a BA.5-related exposure, has not been directly compared with JN.1, representing a BA.2.86-derived exposure, using paired neutralization, antigenic cartography, and longitudinal BCR repertoire profiling from the same individuals.

The regional contrast between BF.7 and JN.1 therefore provided a focused opportunity to examine how infecting-variant antigenicity shapes immune recall. Comparing a BA.5-related exposure with a BA.2.86-derived exposure enabled us to distinguish generalized antibody boosting from humoral updating within Omicron antigenic space. By integrating paired plasma neutralization, antigenic cartography, and longitudinal BCR repertoire profiling, we linked humoral remodeling to changes in the underlying circulating antibody repertoire. We asked whether BF.7 and JN.1 infections elicit equivalent recall responses or divergent humoral trajectories, and whether JN.1 exposure preferentially redirects antibody recognition toward BA.2.86/JN.1-related variants.

## Materials and methods

### Study population and sample collection

Between February and April 2024, individuals with SARS-CoV-2 infection were recruited at Mengchao Hepatobiliary Hospital of Fujian Medical University. Infection was confirmed by RT-PCR, and viral lineages were determined by sequencing. A total of 15 JN.1-infected and 18 BF.7-infected individuals were initially identified; 10 participants from each group were included in the final analysis based on successful lineage assignment and availability of paired samples. Demographic characteristics, clinical severity, symptom duration, hospitalization status, antiviral treatment, vaccination history, prior SARS-CoV-2 infection history, comorbidities, smoking, and alcohol-use information were collected from medical records or participant questionnaires and summarized for baseline comparison between the BF.7 and JN.1 cohorts. In this study, breakthrough infection was defined as sequencing-confirmed BF.7 or JN.1 infection after prior SARS-CoV-2 antigen exposure, including COVID-19 vaccination or prior COVID-19 history. Three unvaccinated BF.7 participants reported prior clinically diagnosed COVID-19 without virological confirmation.

Inclusion criteria were RT-PCR-confirmed SARS-CoV-2 infection with sequencing-confirmed assignment to BF.7 or JN.1. Exclusion criteria were incomplete paired samples, unsuccessful lineage determination, incomplete clinical or vaccination records, known immunodeficiency or ongoing immunosuppressive therapy, and prior receipt of antibody-based treatment. For longitudinal analyses, the earliest available infection-stage sample and the matched convalescent sample from the same participant were treated as paired early- and convalescent-time-point specimens.

Early infection-phase samples were defined as the earliest available blood specimens collected after diagnosis or symptom onset, and convalescent samples were collected 15–30 days after infection. Plasma was used for pseudovirus neutralization assays, and whole blood was used for bulk BCR repertoire sequencing.

### Viral RNA detection, sequencing, and lineage assignment

SARS-CoV-2 infection was confirmed using RT-PCR testing of clinical respiratory specimens according to local diagnostic procedures. For samples with sufficient viral RNA, viral genome sequencing was performed to determine the infecting lineage. Consensus sequences were analyzed for lineage-defining mutations and assigned to BF.7 or JN.1 according to standard Pango SARS-CoV-2 lineage nomenclature (12). Only participants with unambiguous sequencing-supported lineage assignment and paired blood samples were retained in the final analysis.

### Phylogenetic, spike mutation, and epidemic trend analyses

To define the evolutionary and epidemiological context of BF.7 and JN.1, representative SARS-CoV-2 genome sequences and phylogenetic information were retrieved from Nextstrain together with GISAID-derived metadata (13, 14). The phylogenetic tree was recolored to highlight major Omicron lineages and variants used in this study, including BA.2, BA.4/5, BF.7, BQ.1.1, XBB-derived lineages, BA.2.86, JN.1, KP.2, and other variants. Spike mutation patterns and lineage frequencies were analyzed using CoV-Spectrum with GISAID sequence data (15). Mutations with a frequency greater than 10% within the corresponding lineage were included in the mutation summary. Variant frequency curves were generated from time-resolved lineage counts to illustrate epidemic transitions and the relative circulation of BF.7 and JN.1 during the study period.

### Cell culture

293T and Vero E6 cells were obtained from ATCC and maintained in Dulbecco’s modified Eagle’s medium (DMEM; 11965-092, Gibco, Texas, USA) supplemented with 10% fetal bovine serum (FBS), 100 U/mL penicillin, and 100 μg/mL streptomycin according to the supplier’s instructions. I1 mouse hybridoma cells (anti-VSV-G; ATCC, Cat. no. CRL-2700) were cultured in Eagle’s Minimum Essential Medium (EMEM; ATCC, Cat. no. 30-2003) supplemented with 20% FBS, and the culture supernatant was used to neutralize residual VSV-G-pseudotyped ΔG-luciferase virus. All cells were cultured at 37 °C in an incubator with 5% CO_2_. All cell lines were routinely tested for mycoplasma and maintained mycoplasma-free.

### Spike-expression plasmids and pseudovirus panel

Full-length SARS-CoV-2 spike-expression plasmids were generated for the ancestral D614G reference and representative Omicron sublineages included in the neutralization panel as previously described (16). The panel comprised BA.2, BA.4/5, BF.7, BQ.1.1, XBB, XBB.1.5, XBB.1.16, EG.5.1, HK.3, BA.2.86, JN.1, and KP.2, covering major antigenic branches of Omicron, including BA.2/BA.5-related variants, XBB-derived lineages, and the BA.2.86/JN.1-related branch. Spike sequences were designed to contain the lineage-defining substitutions of each variant, enabling comparison of neutralization sensitivity and antigenic relationships across evolutionarily and antigenically distinct Omicron lineages, consistent with previous Omicron pseudovirus neutralization studies (8).

### Production of pseudoviruses

SARS-CoV-2 spike-pseudotyped viruses were generated using a VSV-based ΔG-luciferase system as previously described (6). Plasmids encoding the spikes of SARS-CoV-2 and SARS-CoV-2 variants, as well as spikes carrying single or combined mutations, were synthesized. 293T cells were grown to 3 × 10^6^ cells/mL before transfection with the indicated spike-expression plasmid using polyethylenimine. Cells were cultured overnight at 37 °C with 8% CO_2_, and VSV-G-pseudotyped ΔG-luciferase virus (G*ΔG-luciferase; Kerafast) was then used to infect the cells in DMEM at a multiplicity of infection of 5 for 4 h. Cells were washed three times with PBS containing 2% FBS. The next day, transfection supernatants were collected and clarified by centrifugation at 300 × g for 10 min. Each viral stock was incubated with 20% I1 hybridoma supernatant (anti-VSV-G; ATCC, CRL-2700) for 1 h at 37 °C to neutralize contaminating VSV-G-pseudotyped ΔG-luciferase virus before titration, aliquoting, and storage at -80 °C.

### Pseudovirus neutralization

Neutralization assays were performed by incubating pseudoviruses with serial dilutions of plasma samples and quantifying the reduction in luciferase reporter expression as described previously (17). In brief, Vero E6 cells were seeded in 96-well plates at 2 × 10^4^ cells per well. On the following day, pseudoviruses were incubated with serial dilutions of test samples in triplicate for 30 min at 37 °C. The mixtures were added to cultured cells and incubated for an additional 24 h. Luminescence was measured using the Luciferase Assay System (Beyotime). ID_50_ was defined as the dilution at which relative light units were reduced by 50% compared with virus-control wells (virus plus cells) after subtraction of background signal from cell-only control wells. ID_50_ values were calculated by nonlinear regression in GraphPad Prism.

### Antigenic cartography

Antigenic maps were constructed from plasma neutralization ID_50_ matrices using the Racmacs package in R, following the classical antigenic cartography framework (18). Maps were generated in two-dimensional space using 10, 000 optimization steps and default parameters. The positions of viral antigens and plasma samples were optimized so that map distances reflected fold decreases in neutralizing ID_50_ titers relative to the maximum titer for each plasma sample. One antigenic map unit represents a two-fold change in ID_50_ titer. Separate early- and convalescent-time-point maps were generated for the BF.7 and JN.1 cohorts, and a combined map was used to compare overall antigenic reorganization after infection.

### Bulk BCR repertoire sequencing and analysis

Whole-blood samples collected at paired early and convalescent time points were subjected to bulk BCR repertoire sequencing. Immunoglobulin variable-region libraries were prepared and sequenced on the Illumina NovaSeq PE150 platform, and raw sequencing data were obtained in FASTQ format. Productive BCR sequences were analyzed for V(D)J assignment, CDR3 identification, and clonotype calling using a standard immunorepertoire pipeline.

After sequencing quality control, one participant was excluded because sequencing data were not successfully generated, and another was excluded because of abnormal repertoire data that did not meet downstream analysis criteria. Bulk BCR analyses were therefore performed on 18 participants with paired samples, including 9 BF.7-infected and 9 JN.1-infected individuals. After removal of duplicate entries, repertoire features, including V/J gene usage, CDR3 length distribution, clonotype abundance, and repertoire diversity, were summarized for each participant and time point. Longitudinal changes were assessed using paired early and convalescent samples, and group-level differences were compared between the BF.7 and JN.1 cohorts. Productive IGH reads were used to evaluate total IGH reads, clonotype counts, Shannon-Wiener diversity, IGHV/IGHJ usage, and IGHV-IGHJ pairing. Productive IGK and IGL sequences were used to analyze light-chain diversity, V/J usage, V-J pairing, CDR3 length, and sequence-logo features when available. Violin plots, family-usage bar plots, stacked V-gene composition plots, chord diagrams, density plots, and sequence logos were generated to visualize BCR repertoire features.

### Statistical analysis

Data were analyzed using GraphPad Prism 8.0. Paired comparisons were performed using the Wilcoxon signed-rank test, between-group comparisons using the Mann-Whitney U test, and correlations using Spearman’s rank correlation test. Exploratory age-correlation analyses were performed for selected neutralization and IGH repertoire endpoints. No formal power calculation or prespecified effect-size estimation was performed. For analyses involving multiple related comparisons, Benjamini–Hochberg false-discovery-rate correction was applied within predefined comparison families, including lineage-stratified neutralization panels and BCR repertoire feature categories. FDR-adjusted q values < 0.05 were considered multiplicity-adjusted evidence. Given the exploratory cohort size, P values were interpreted descriptively, and adjusted or subgroup analyses were not performed.

### Ethics approval

This study was approved by the Ethics Committee of Fujian Medical University Mengchao Hepatobiliary Hospital (approval no. 2024_052_01). Written informed consent was obtained from all participants before sample collection and analysis.

## Results

### Evolutionary divergence, spike mutational patterns, and epidemic positioning of BF.7 and JN.1

**Figure 1:**
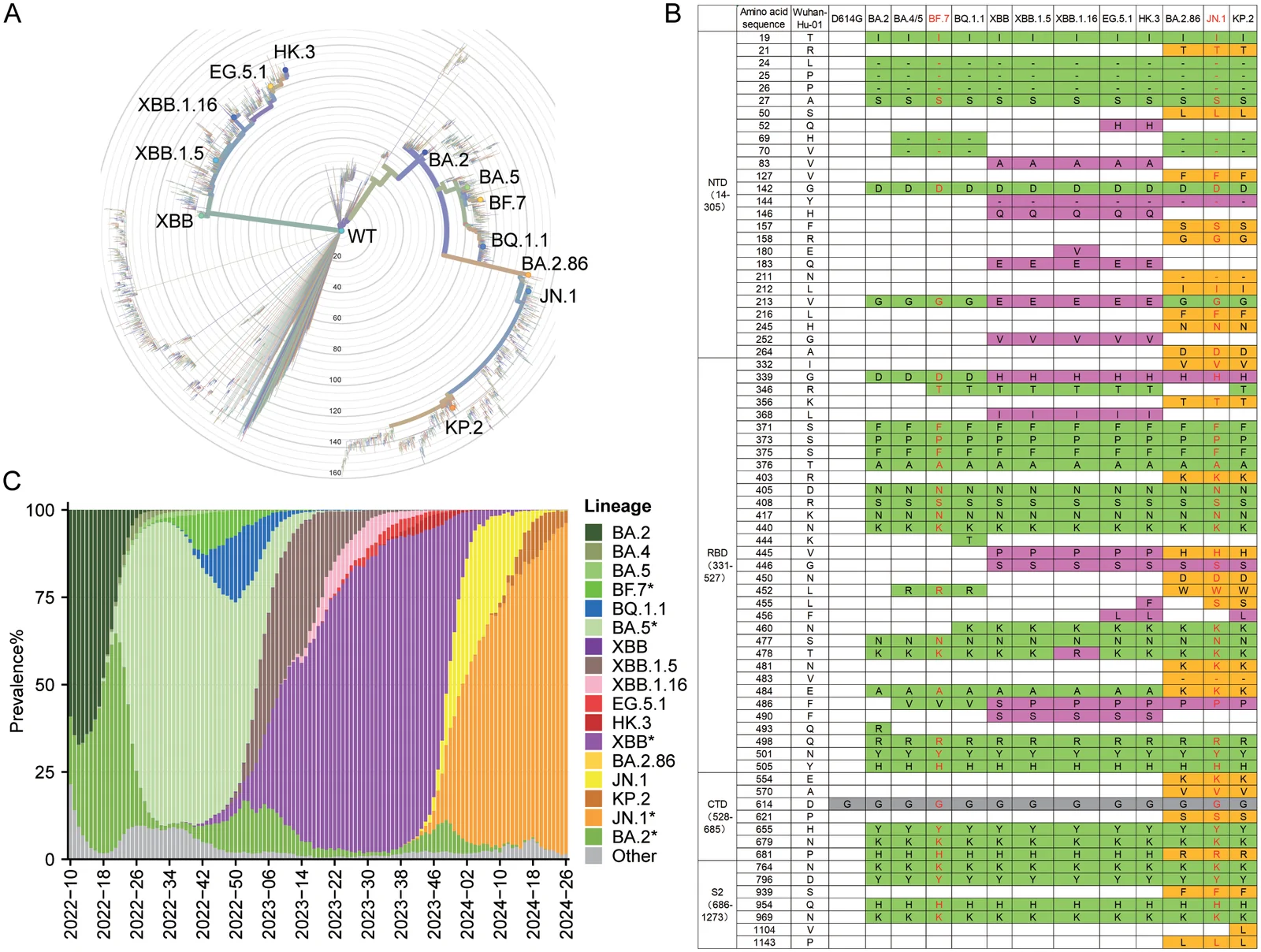
Evolutionary divergence and epidemic positioning of BF.7 and JN.1. **(A)** Phylogenetic tree showing the evolutionary relationships among representative SARS-CoV-2 variants, including BF.7, BA.2.86, JN.1, KP.2, and XBB-related lineages. **(B)** Spike mutation map of representative SARS-CoV-2 variants. Amino acid substitutions are shown across the spike protein, including the N-terminal domain, receptor-binding domain, C-terminal domain, and S2 region. **(C)** Temporal prevalence of SARS-CoV-2 variants from 2022 to 2024, showing the transition from earlier Omicron lineages to later JN.1-related variants. In **(C)** asterisks denote the corresponding lineage and its sublineages, with separately labeled variants excluded from the grouped categories; Other denotes all remaining variants not shown as individual or grouped lineages.

To establish the virological basis for comparing BF.7 and JN.1 infections, we first assessed whether the two infecting lineages occupied distinct evolutionary and molecular backgrounds within Omicron. We integrated phylogenetic relatedness, spike mutational composition, and epidemic timing to define the variant-level context before analyzing plasma neutralization and BCR repertoire remodeling. To this end, we performed Nextclade-based phylogenetic reconstruction of major Omicron lineages distributed across divergent radial branches (19). BF.7 was positioned within the BA.4/5-related branch, near BA.5-descended variants such as BQ.1.1, whereas JN.1 clustered within the BA.2.86-derived branch, close to later descendants such as KP.2 (**Figure 1A**). This topology indicated that BF.7 and JN.1 arose through distinct evolutionary routes rather than representing adjacent variants within the same Omicron background. Spike mutation profiling further showed that this phylogenetic separation was accompanied by marked differences in antibody-exposed regions (**Figure 1B**). Of note, BF.7 carried R346T, a recurrent immune-evasion-associated substitution, whereas JN.1 harbored L455S and multiple BA.2.86-derived substitutions across the N-terminal domain and receptor-binding domain. These differences support the classification of BF.7 and JN.1 as antigenically differentiated exposures.

Moreover, epidemic trend analysis placed the two lineages in different phases of Omicron succession (**Figure 1C**). BF.7 was associated with an earlier BA.5-related phase, whereas JN.1 became prominent during the later BA.2.86-derived phase. Regional detection of both lineages during the study period provided a real-world comparison between an earlier BA.5-related lineage and a later BA.2.86-derived lineage. Together, these phylogenetic, mutational, and epidemiological data establish BF.7 and JN.1 as distinct immune-shaping exposures and provide the foundation for assessing divergent neutralization, antigenic, and BCR repertoire trajectories.

### Baseline characteristics of the study participants

Having established that BF.7 and JN.1 represented distinct viral exposures, we next evaluated the demographic and immunological context of the two infection cohorts. Because neutralization and BCR repertoire differences can be shaped by age, vaccination history, prior exposure interval, comorbidities, and sampling time, we summarized demographic, clinical, and vaccination characteristics of the final cohort before functional immune profiling (**Table 1**).

**Table 1:** Baseline characteristics of enrolled participants.

Characteristic		BF.7 breakthrough infection individuals (n=10)	JN.1 breakthrough infection individuals (n=10)
Age(years), median(range)		47.5 (25–90)	70.0(29-86)
Male, n(%)		6(60)	4(40)
BMI(kg/m2), mean(SD)		24.2(2.7)	22.6(1.9)
Number of COVID-19 vaccinations, median(range)		3.0(0-4)*	3.0(0-3)
times of COVID-19 infection, median(range)		1(0-1)*	1.0(0-1)
Days since the last COVID-19 vaccination or breakthrough infection to the current breakthrough infection, median(range)		445.0(271-837)	429.0(272-738)
Days from symptom onset to first sample collection, median(range)		2.0(0-3)	1.0(0-3)
Days from symptom onset to second sample collection, median(range)		15.0(13-19)	16.0(13-26)
Disease severity, n (%)†	Mild	9 (90)	10 (100)
	Moderate (with pneumonia)	1 (10)	0 (0)
Comorbidities, n(%)	Any	6(60)	5(50)
	Diabetes	2(20)	0(0)
	Hypertension	1(10)	2(20)
	Cardiac Disease	0(0)	2(20)
	Chronic lung disease	1(10)	1(10)
	Cerebrovascular disease	1(10)	0(0)
	Chronic kidney disease	0(0)	0(0)
	Cancer	1(10)	0(0)
	Smoking	2(20)	2(20)
	Alcohol use	2(20)	2(20)
	Illicit drug use	0(0)	0(0)
	Autoimmune disorder	0(0)	1(10)

*Three participants in the BF.7 cohort had no prior COVID-19 vaccination record but reported prior clinically diagnosed COVID-19 without virological confirmation. They were therefore retained under the study definition of breakthrough infection, and uncertainty in prior infection documentation is acknowledged in the Discussion.

†Disease severity was classified according to the Diagnosis and Treatment Protocol for COVID-19 (China Trial Version 10). All patients received symptomatic outpatient care; no antiviral therapy or hospitalization was required.

The final cohort comprised 20 participants with sequencing-confirmed infection, including 10 BF.7-infected and 10 JN.1-infected individuals. The JN.1 cohort was older, with a median age of 70.0 years compared with 47.5 years in the BF.7 cohort. Exploratory correlation analyses did not identify significant associations between age and selected neutralization or IGH repertoire endpoints (**Supplementary Table 1**). Apart from this age difference, the two cohorts were broadly similar in sex distribution, vaccination burden, prior exposure interval, sampling schedule, and overall comorbidity burden. The median number of COVID-19 vaccine doses was 3.0 in both cohorts, although the BF.7 cohort showed a broader distribution, including three participants who were unvaccinated but had prior clinically diagnosed COVID-19 without virological confirmation. The interval from the last COVID-19 vaccination or prior infection to the current infection and the sampling schedules were comparable between cohorts. Overall comorbidity burden was also similar, and smoking and alcohol use were reported in 20% of participants in each group. These baseline features support paired within-person analyses while indicating that age and prior exposure history should be considered when interpreting between-group differences.

### Differential neutralization after BF.7 and JN.1 breakthrough infection

To determine how these two antigenically distinct infections altered circulating antibody function, we measured paired early- and convalescent-phase plasma neutralization against a broad pseudovirus panel. This panel included D614G as an ancestral reference and multiple Omicron sublineages spanning BA.2/BA.5-related viruses (BA.2, BA.4/5, BF.7, and BQ.1.1), XBB-derived variants (XBB, XBB.1.5, XBB.1.16, EG.5.1, and HK.3), and BA.2.86/JN.1-related viruses (BA.2.86, JN.1, and KP.2). This design enabled us to distinguish generalized increases in neutralizing activity from variant- or lineage-biased boosting.

**Figure 2:**
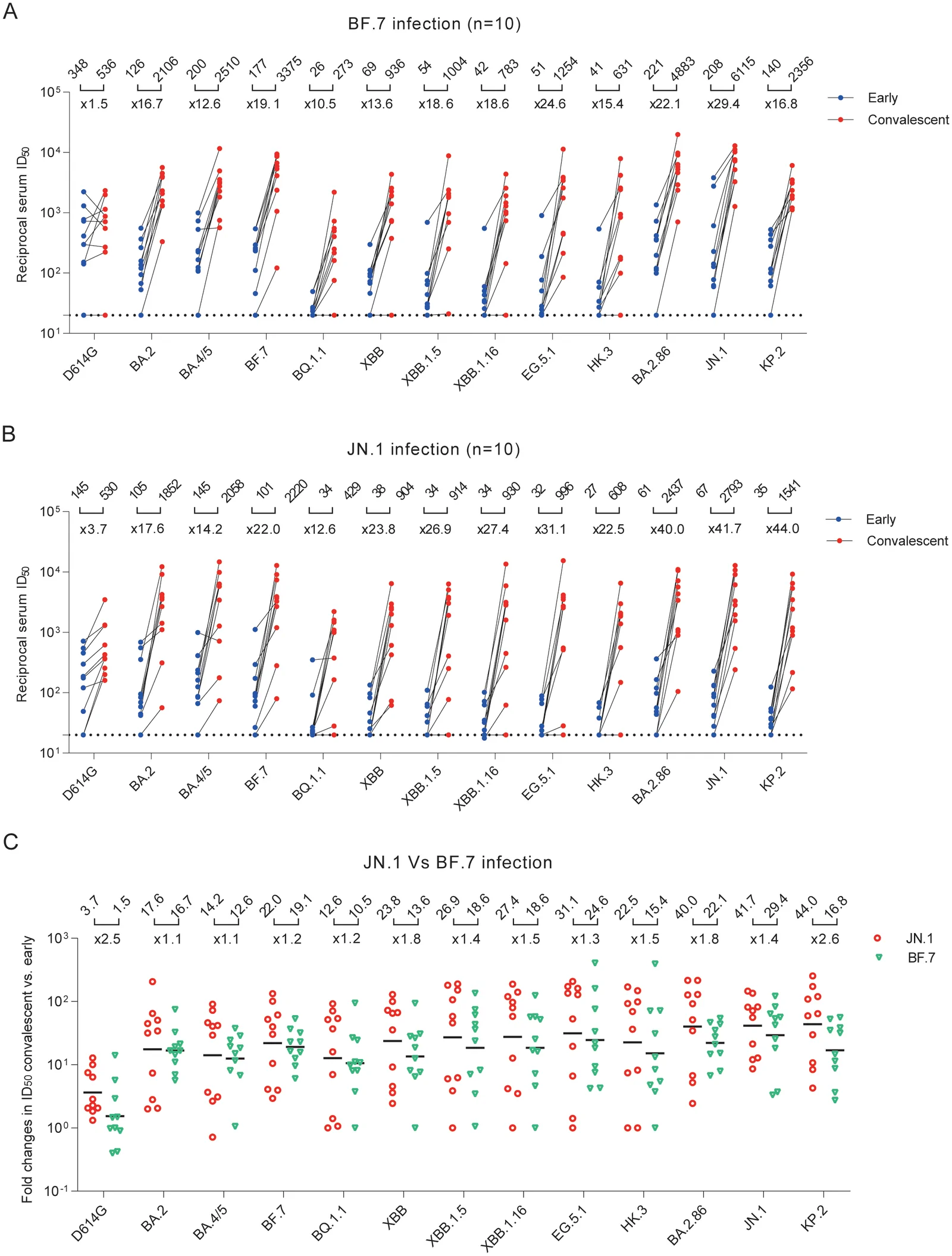
Variant-dependent neutralization boosting after BF.7 and JN.1 breakthrough infection. **(A)** Neutralizing antibody titers against the indicated pseudoviruses in paired early- and convalescent-phase plasma samples from the BF.7 cohort. **(B)** Neutralizing antibody titers against the same pseudovirus panel in paired early- and convalescent-phase plasma samples from the JN.1 cohort. **(C)** Comparison of convalescent-to-early fold changes in neutralizing titers between the BF.7 and JN.1 cohorts. Neutralizing antibody titers are shown as reciprocal ID_50_ values. Numbers above the plots indicate group-level titers and fold changes as shown.

We found that both BF.7 and JN.1 infections boosted neutralization across all tested variants, but the magnitude and direction of boosting differed (**Figure 2**). BF.7 infection enhanced recognition even of antigenically advanced Omicron variants, with the largest convalescent-to-early fold increases observed against EG.5.1 (24.6-fold), BA.2.86 (22.1-fold), JN.1 (29.4-fold), and KP.2 (16.8-fold) (**Figure 2A**; **Supplementary Figure 1**). By contrast, JN.1 infection elicited a larger and more late-Omicron-oriented response, with fold increases of 40.0-fold against BA.2.86, 41.7-fold against JN.1, and 44.0-fold against KP.2, along with robust boosting against EG.5.1 (31.1-fold), XBB.1.16 (27.4-fold), and XBB.1.5 (26.9-fold) (**Figure 2B**; **Supplementary Figure 2**). Moreover, direct comparison of convalescent-to-early fold changes further showed that the JN.1 cohort generally mounted larger increases than the BF.7 cohort, most prominently against BA.2.86 (1.8-fold) and KP.2 (2.6-fold), as well as several XBB-derived variants, including XBB (1.8-fold) (**Figure 2C**). These results indicate that the functional outcome of infection was shaped by the antigenic identity of the infecting variant rather than reflecting a uniform Omicron recall response. Thus, although both infections increased pre-existing humoral activity, their effects were exposure-dependent: BF.7 broadened neutralization from a BA.5-related background, whereas JN.1 drove a stronger late-Omicron-oriented response.

### Lineage-biased Omicron neutralization after BF.7 and JN.1 breakthrough infection

Because fold-change analysis showed broad boosting but did not resolve whether all Omicron branches were neutralized equally after infection, we next performed a lineage-stratified analysis of late-convalescent plasma. Variants were grouped as BA.2/BA.5-related viruses, XBB-derived lineages, and the BA.2.86/JN.1-related branch. This analysis tested whether infection erased pre-existing antigenic boundaries or whether escape remained structured within specific Omicron branches.

**Figure 3:**
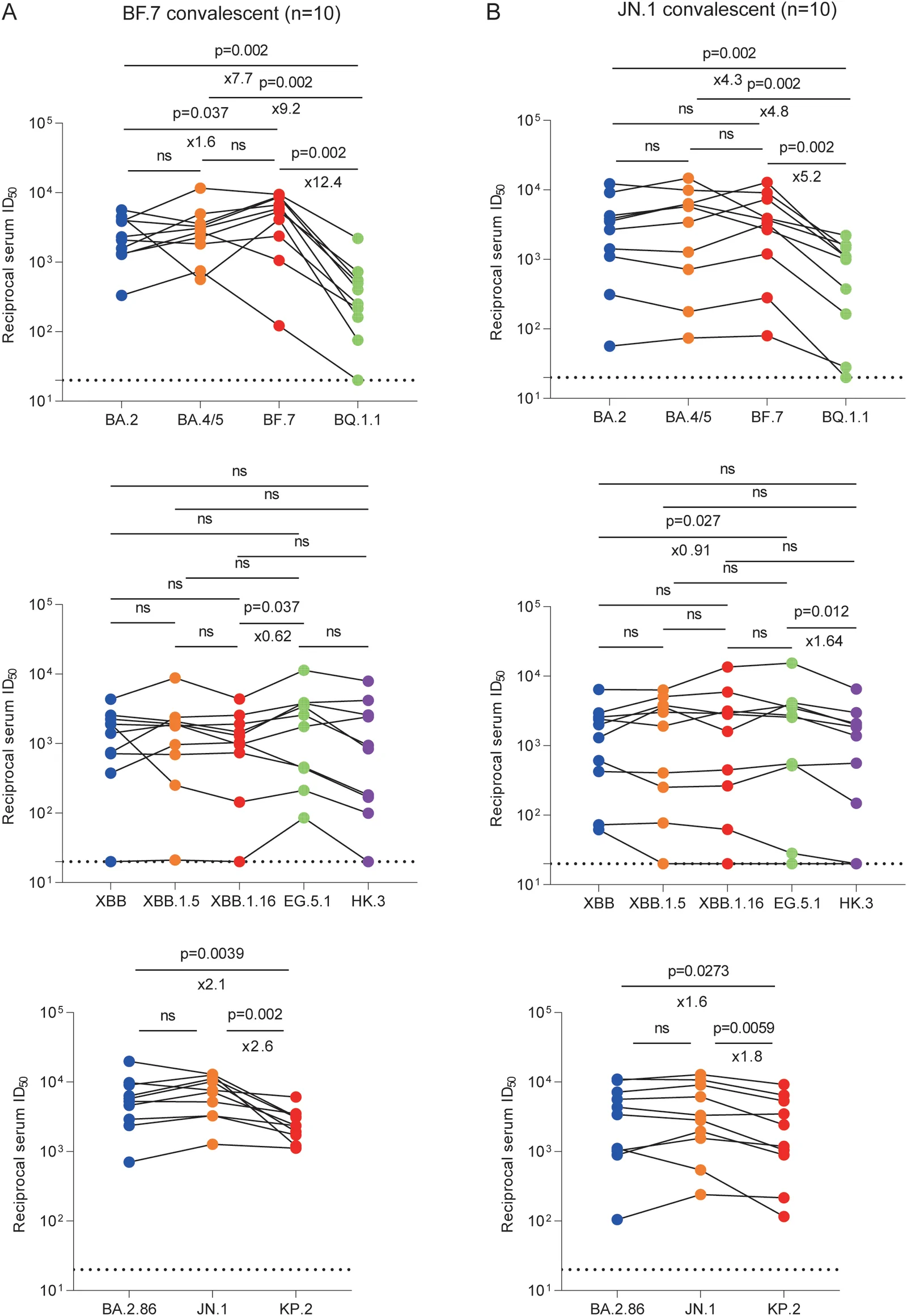
Branch-specific neutralization hierarchy after BF.7 and JN.1 breakthrough infection. **(A)** Neutralizing antibody titers of BF.7 convalescent plasma against BA.2/BA.5-related variants, XBB-derived variants, and BA.2.86/JN.1-related variants. **(B)** Neutralizing antibody titers of JN.1 convalescent plasma against the same lineage-stratified pseudovirus groups. Neutralizing antibody titers are shown as reciprocal ID_50_ values. Lines connect titers from the same participant, and dotted lines indicate the lower limit of detection.

Late-convalescent plasma samples were analyzed against lineage-stratified Omicron pseudovirus groups, including BA.2/BA.5-related variants, XBB-derived lineages, and BA.2.86/JN.1-related viruses (**Figure 3**). Across both cohorts, a consistent hierarchy emerged in which BQ.1.1 and KP.2 were the least sensitive viruses within the BA.2/BA.5-related and BA.2.86/JN.1-related branches, respectively. In BF.7 late-convalescent plasma, BA.2, BA.4/5, and BF.7 were neutralized at broadly similar levels, whereas BQ.1.1 was markedly less sensitive, with ID_50_ titers reduced by 7.7-, 9.2-, and 12.4-fold relative to BA.2, BA.4/5, and BF.7, respectively. Neutralization of XBB-derived variants was comparatively preserved, with modest within-branch variation and no single dominant escape outlier. Within the BA.2.86/JN.1-related branch, BA.2.86 and JN.1 showed similar sensitivity, whereas KP.2 was less efficiently neutralized, with titers 2.1- and 2.6-fold lower than those against BA.2.86 and JN.1, respectively (**Figure 3A**). A similar hierarchy was observed in JN.1 late-convalescent plasma: BQ.1.1 remained less sensitive than BA.2, BA.4/5, and BF.7, with 4.3-, 4.8-, and 5.2-fold lower titers, respectively, whereas KP.2 was 1.6- and 1.8-fold less sensitive than BA.2.86 and JN.1 (**Figure 3B**). This lineage-stratified analysis refines the interpretation of **Figure 2** by showing that infection increased neutralization breadth while preserving variant-specific escape profiles. Post-infection plasma therefore showed broader neutralization but remained branch-structured, providing the rationale for the antigenic cartography analysis that follows. These lineage-leading escape patterns, particularly those involving BQ.1.1 and KP.2, remained evident after adjustment for multiple comparisons (**Supplementary Tables 2**, **S3**).

### Antigenic maps of BF.7 and JN.1 breakthrough infection

**Figure 4:**
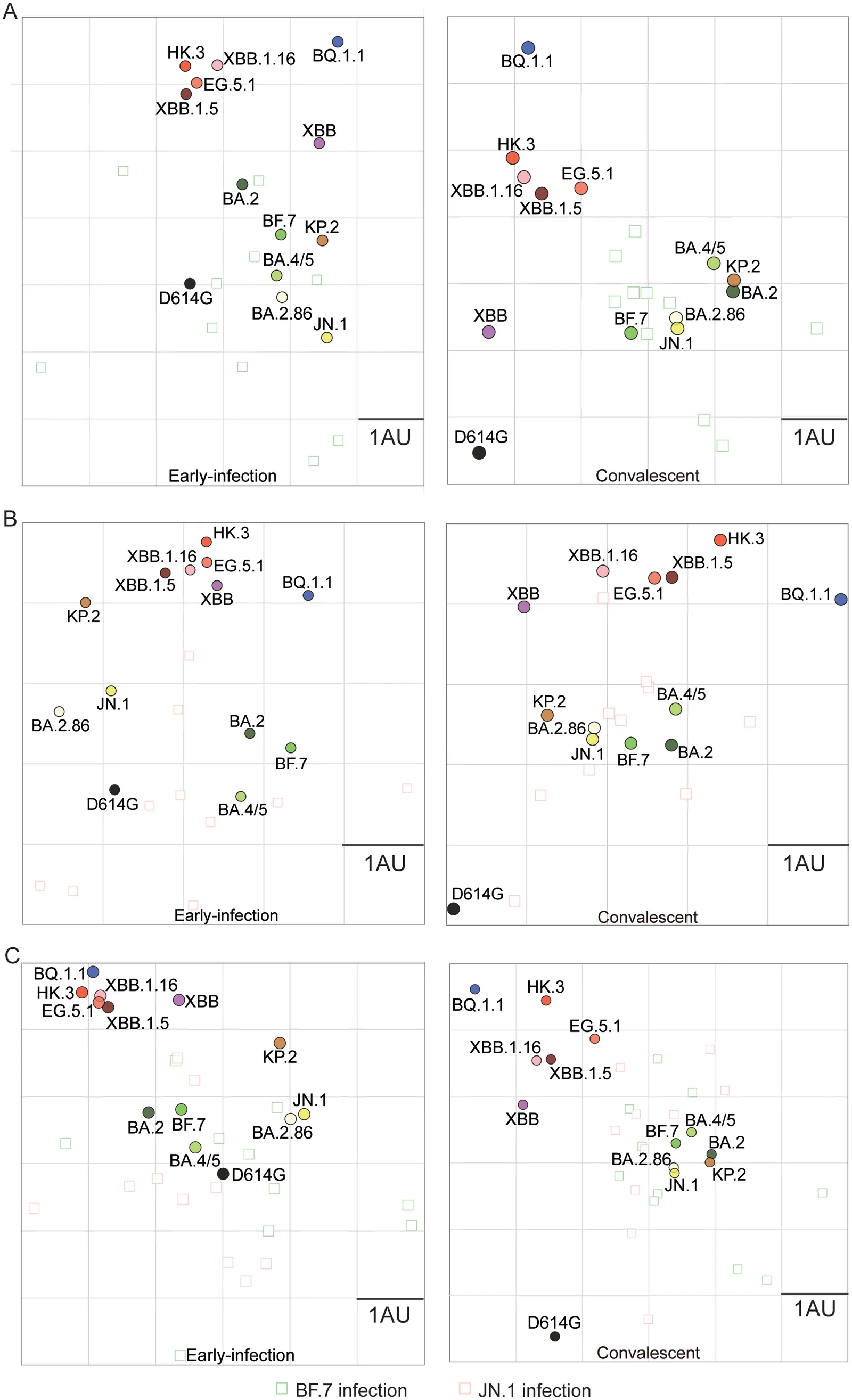
Antigenic maps of Omicron variants after BF.7 and JN.1 breakthrough infection. **(A)** Antigenic maps of early- and convalescent-phase plasma from the BF.7 cohort. **(B)** Antigenic maps of early- and convalescent-phase plasma from the JN.1 cohort. **(C)** Combined antigenic maps of the BF.7 and JN.1 cohorts. Filled circles represent viral antigens, and open squares represent plasma samples. Green and pink squares indicate BF.7 and JN.1 samples, respectively. One antigenic unit represents a two-fold change in neutralization titer.

To move beyond individual titer comparisons and determine how infection reorganized antigenic relationships among variants, we reconstructed antigenic maps from the neutralization data (**Figure 4**). In this framework, map distance reflects the fold loss of plasma neutralization, with one antigenic unit corresponding to a two-fold change in ID50. Viruses positioned closer together are therefore recognized more similarly by the same plasma samples, whereas greater separation indicates antigenic divergence or escape. This analysis tested whether post-infection plasma broadly reduced antigenic separation across Omicron variants or preferentially reshaped relationships within specific variant branches.

At the early time point, BA.2/BA.5-related variants, XBB-derived lineages, and BA.2.86/JN.1-related viruses occupied distinct antigenic regions in both cohorts, indicating that pre-existing plasma still recognized major Omicron branches as separable groups and providing a baseline for the recall response. After BF.7 infection, the convalescent map showed closer positioning of BA.2, BA.4/5, BF.7, BA.2.86, JN.1, and KP.2, consistent with the broad boosting observed in neutralization assays; however, BQ.1.1 and XBB-derived variants remained relatively separated (**Figure 4A**). In contrast, JN.1 infection produced a comparable but more late-Omicron-oriented reorganization, with BA.2.86, JN.1, and KP.2 aligning more closely with BA.2, BA.4/5, and BF.7, while BQ.1.1 and the XBB branch continued to occupy distinct positions (**Figure 4B**). Furthermore, the combined cohort map recapitulated these patterns, showing preferential reduction of antigenic distances among BA.2/BA.5-related and BA.2.86/JN.1-related viruses without full convergence of BQ.1.1 or XBB-derived variants (**Figure 4C**). These cartographic results extend the lineage-stratified neutralization analysis by showing that infection did not simply raise titers uniformly. Instead, it selectively reshaped antigenic space by drawing related Omicron branches closer together while leaving other escape trajectories relatively distinct. The humoral updating after BF.7 or JN.1 infection is therefore best characterized as selective convergence of related antigenic neighborhoods rather than complete antigenic homogenization.

### BCR repertoire remodeling after BF.7 and JN.1 breakthrough infection

**Figure 5:**
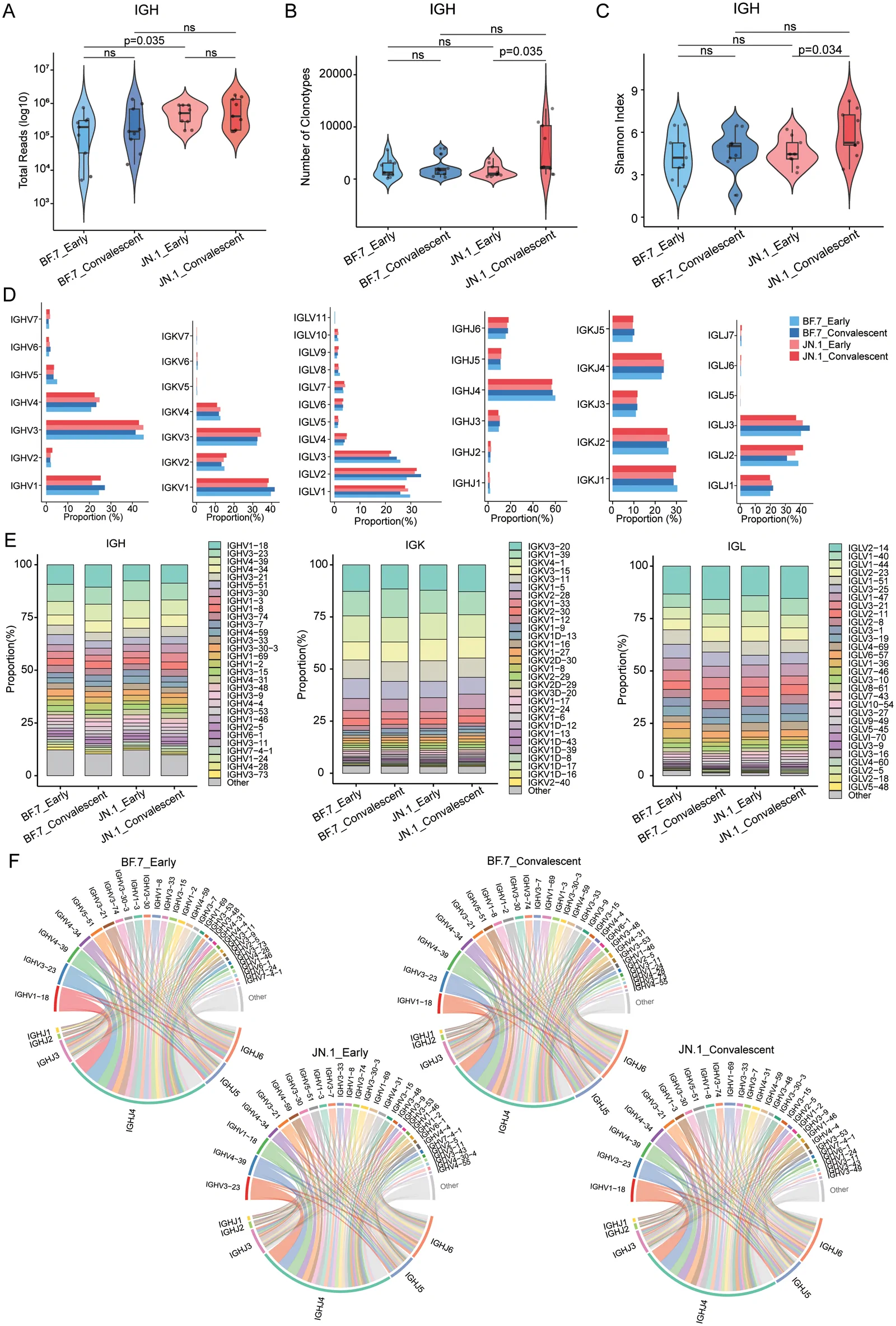
Bulk BCR repertoire profiles after BF.7 and JN.1 breakthrough infection. **(A)** IGH read counts in paired early and convalescent samples. **(B)** IGH clonotype counts. **(C)** Shannon diversity of the IGH repertoire. **(D)** V- and J-family usage across IGH, IGK, and IGL repertoires. **(E)** V-gene usage frequencies in productive IGH, IGK, and IGL repertoires. **(F)** IGHV-IGHJ pairing architecture shown by chord diagrams. Bulk BCR repertoires were analyzed in paired early and convalescent whole-blood samples from BF.7- or JN.1-infected individuals.

Bulk BCR sequencing of paired early and convalescent whole-blood samples was used to assess population-level repertoire remodeling after BF.7 and JN.1 infection, without assigning antigen specificity or neutralizing activity to individual clonotypes. In this section, clonotype count is used as a measure of repertoire richness, Shannon diversity as an index incorporating both richness and clonal evenness, and repertoire remodeling as paired early-to-convalescent changes in clonotype diversity, V/J gene usage, isotype composition, and within-chain V–J gene pairing. After quality control, paired repertoires from 18 participants were retained for downstream analysis, including 9 BF.7-infected and 9 JN.1-infected individuals. IGH read counts remained broadly comparable between early and convalescent samples in both cohorts, indicating that post-infection repertoire changes were not primarily attributable to a uniform increase in heavy-chain sequencing abundance (**Figure 5A**). In contrast, IGH clonotype counts and Shannon diversity showed nominal early-to-convalescent increases in the JN.1 cohort, with less evident change after BF.7 infection (**Figures 5B, C****;** **Supplementary Table 4**). This pattern was consistent with broader heavy-chain repertoire-level changes after JN.1 infection.

Family-level analysis placed this increase in repertoire complexity within a largely preserved germline framework (**Figure 5D**). IGHV1, IGHV3, and IGHV4 remained the major heavy-chain V-family compartments, and IGHJ4 remained the dominant J family across cohorts and time points. Directional changes were not distributed evenly across the repertoire but were concentrated along selected family axes. In the heavy-chain compartment, shifts involving IGHV4, IGHV5, and IGHV7 were accompanied by changes in IGHJ3 and IGHJ4, with additional signals in the lower-frequency IGHJ1 and IGHJ2 families. Light-chain usage was more constrained, with the dominant IGKV, IGLV, IGKJ, and IGLJ hierarchies largely maintained. Light-chain signals mainly involved proportional changes around IGKV2 in the kappa V compartment; IGLV1, IGLV3, and IGLV4 in the lambda V compartment; and selected J-family shifts including IGKJ4, IGLJ2, IGLJ3, and IGLJ6. This pattern was consistent with selective reweighting of existing germline-family usage, with broader redistribution after JN.1 infection.

The same repertoire pattern was evident at the individual V-gene level, where dominant IGH, IGK, and IGL V-gene components were retained but proportionally reweighted from early to convalescent sampling (**Figure 5E**). In the IGH repertoire, high-ranking genes such as IGHV1-18, IGHV3-23, IGHV4-39, IGHV4-34, and IGHV3–21 remained prominent across all four groups, indicating preservation of the major heavy-chain architecture. The relative contributions of these dominant genes nevertheless shifted after infection, with BF.7-associated changes appearing more focused and JN.1-associated changes extending across a broader set of IGHV components. Light-chain repertoires showed a more stable structure. IGKV3-20, IGKV1-39, IGKV4-1, IGKV3-15, and IGKV3–11 continued to account for a large fraction of the IGK repertoire, while IGLV2-14, IGLV1-40, IGLV1-44, and IGLV2–23 remained among the major IGL components. These findings support a model in which infection altered the weighting of pre-existing V-gene components rather than generating broad replacement of the heavy- or light-chain repertoire. Related light-chain repertoire features are summarized in **Supplementary Figure 3**, with additional isotype and light-chain CDR3 analyses shown in **Supplementary Figure 4**.

Preservation of dominant germline usage was further reflected in IGHV-IGHJ pairing architecture (**Figure 5F**). Chord diagrams showed that the major recombination framework persisted across early and convalescent samples in both cohorts, with recurrent IGHV families continuing to pair predominantly with IGHJ4. The principal post-infection change was therefore a redistribution of connection weights within an existing pairing structure. BF.7 infection produced a more concentrated reweighting pattern, whereas JN.1 infection showed a more broadly distributed shift across major heavy-chain pairing connections, in line with its stronger repertoire diversification.

**Figure 6:**
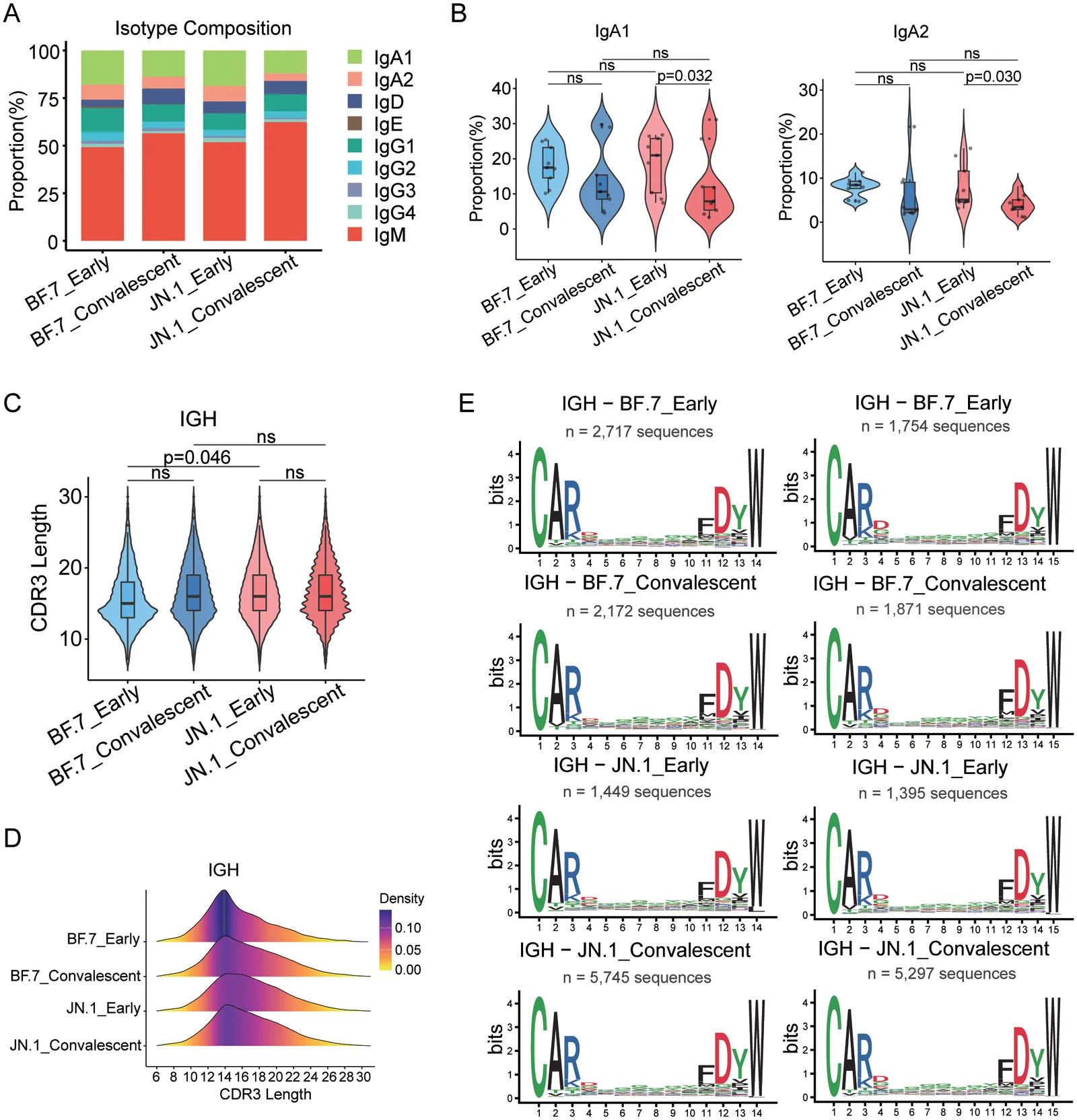
Isotype composition and IGH CDR3 features after BF.7 and JN.1 breakthrough infection. **(A)** Isotype and subclass composition of productive BCR repertoires. **(B)** Paired comparison of IgA1 and IgA2 proportions. **(C)** IGH CDR3 length distributions. **(D)** Density profiles of IGH CDR3 length. **(E)** Representative IGH CDR3 sequence-logo profiles. Samples are grouped by infecting variant and sampling time point.

Changes in repertoire composition were also accompanied by variant-associated differences in antibody class usage (**Figures 6A, B**). IgM represented the largest isotype fraction across all groups, while IgA1 accounted for a substantial additional proportion of the repertoire. IgA2, IgG subclasses, IgD, and IgE contributed smaller fractions. Subclass-level comparison showed nominal IgA1 and IgA2 proportions decreased from early to convalescent sampling in the JN.1 cohort that did not remain significant after FDR correction (P = 0.032 and P = 0.030, respectively; **Supplementary Table 5**), whereas the BF.7 cohort showed no significant paired change (**Figure 6B**). Thus, the clearest isotype-level signal was observed after JN.1 infection, suggesting that broader repertoire remodeling in this cohort also involved changes in antibody class composition.

IGH CDR3 architecture remained comparatively stable despite the observed changes in diversity, gene usage, pairing weights, and isotype composition (**Figures 6C–E**). Paired early-to-convalescent comparisons showed no significant shift in IGH CDR3 length after either BF.7 or JN.1 infection, although BF.7 and JN.1 early samples showed a nominal baseline difference (P = 0.046; **Figure 6C**; **Supplementary Table 6**). Density profiles confirmed that the dominant IGH CDR3 length landscape was maintained after infection, with only subtle redistribution across the main length range (**Figure 6D**). Representative sequence-logo analysis further showed preservation of major terminal CDR3 sequence features across cohorts and time points (**Figure 6E**), arguing against broad CDR3 compositional restructuring. Extended length-stratified CDR3 sequence-logo profiles are shown in **Supplementary Figures 5**–**S7**.

At the repertoire level, BF.7 and JN.1 infections remodeled circulating BCR profiles mainly through selective V- and J-family redistribution, V-gene reweighting, IGHV-IGHJ pairing adjustment, diversity changes, and isotype shifts. This remodeling was more extensive after JN.1 infection and paralleled the stronger late-Omicron-oriented response observed in the serological analyses. Because these data were generated from bulk whole-blood BCR repertoires, they should be interpreted as population-level evidence of repertoire remodeling rather than direct identification of SARS-CoV-2-specific or neutralizing clonotypes.

## Discussion

SARS-CoV-2 has entered a phase of recurrent circulation, in which variant turnover, regional heterogeneity, and repeated immune exposure increasingly shape the population immune landscape (20). In this setting, the key immunological question is no longer simply whether infection boosts antibody responses, but whether antigenically distinct Omicron lineages remodel humoral immunity in different ways. Here, we compared BF.7 and JN.1 infections, two Omicron exposures detected within a shared regional setting but positioned in distinct antigenic backgrounds. Both infections enhanced cross-Omicron neutralization, yet JN.1 infection was associated with a stronger late-Omicron-oriented response and broader BCR repertoire remodeling. These findings suggest that antigenically distinct Omicron infections can shape humoral immune updating along different trajectories, with between-group differences interpreted in light of cohort differences in age and prior immune exposure.

The epidemiological setting of this comparison is informative. National surveillance in China identified JN.1 as the predominant lineage by mid-2024, whereas regional sequencing in Fujian detected BF.7 as well as JN.1 during the study period (3). This regional contrast provided a natural comparison between two Omicron lineages positioned at different stages of antigenic evolution. BF.7 belongs to the BA.5-related branch, whereas JN.1 is a BA.2.86 descendant that emerged after extensive antigenic drift through the XBB era (1, 21). This setting allowed us to examine whether an earlier BA.5-related exposure and a later BA.2.86/JN.1-related exposure were associated with divergent humoral outcomes under real-world conditions. Such direct comparisons remain limited, as most studies of Omicron infection have focused on BA.1-, BA.2-, BA.5-, or XBB-era exposures rather than later lineages separated in antigenic space (9, 10).

The evolutionary relationship between BF.7 and JN.1 provides a plausible basis for the observed difference in neutralization boosting. BF.7 belongs to the BA.5-related branch and represents an earlier Omicron immune-evasion background. In contrast, JN.1 emerged from the BA.2.86-derived lineage after extensive antigenic drift through the XBB era (1, 21, 22). The stronger late-Omicron-oriented response observed after JN.1 infection is consistent with the greater antigenic displacement of this lineage. Exposure to a more antigenically shifted spike may impose stronger selective pressure on pre-existing humoral memory. This may favor broader recall and redistribution of cross-reactive B-cell populations rather than simple amplification of established antibody specificities. This interpretation is consistent with evidence that prior variant exposure conditions subsequent immune boosting and that repeated Omicron exposure can reshape ancestral immune imprinting (5, 23).

The lineage-stratified neutralization results further show that this broadening was structured rather than uniform. In both cohorts, BQ.1.1 remained the least sensitive variant within the BA.2/BA.5-related branch, whereas KP.2 remained relatively resistant within the BA.2.86/JN.1-related branch. Plasma neutralizing activity broadened after infection, but branch-specific escape hierarchies persisted. This pattern is biologically plausible. BQ.1.1 carries convergently selected receptor-binding domain substitutions, including R346T, K444T, N460K, and F486V, that are associated with escape from polyclonal antibody responses and multiple antibody classes (24–26). KP.2 is a later antibody-evasive JN.1 descendant, consistent with evidence that JN.1-derived variants continue to diversify in virological and antibody-evasion phenotypes (27). The persistence of these resistant variants after infection suggests that antigenic broadening does not flatten Omicron antigenic space but instead leaves a structured landscape in which branch-leading escape variants remain immunologically consequential.

The mutational distinction between BF.7 and JN.1 is consistent with this pattern. BF.7 is characterized in part by R346T, a recurrent immune-evasion-associated substitution also found in several Omicron escape lineages. JN.1, by contrast, emerged on the BA.2.86 background and features additional spike remodeling, including L455S, which has been linked to JN.1 virological properties and immune evasion. Although the present study does not isolate the effect of individual substitutions, the neutralization pattern is compatible with the broader BA.2.86/JN.1 spike architecture contributing to a stronger late-Omicron-oriented response after JN.1 infection. This pattern is better interpreted in relation to the antigenic architecture of each lineage than to epidemic timing alone.

Antigenic cartography provided a complementary view of humoral updating and is increasingly used to assess SARS-CoV-2 immune escape and vaccine antigenic mismatch (28). If fold changes alone were considered, infection might appear to simply raise neutralizing titers across variants. However, antigenic maps revealed a more selective reorganization. Early plasma separated BA.2/BA.5-related variants, XBB-derived lineages, and BA.2.86/JN.1-related viruses into distinguishable antigenic regions. After infection, these distances were reduced among related Omicron branches, whereas BQ.1.1 and XBB-derived variants remained relatively distinct. Infection therefore reorganized antigenic space directionally rather than flattening it globally. This pattern supports current models of immune imprinting, in which repeated Omicron exposure can reshape, but does not erase, pre-existing antigenic hierarchy (5, 10, 29).

The BCR repertoire data provide an immunological framework for the serological findings. Infection did not cause a uniform increase in IGH read abundance, whereas JN.1 infection was accompanied by increased clonotype counts and Shannon diversity, indicating broader heavy-chain repertoire complexity rather than simple sequencing-depth variation. The dominant signal was selective reweighting of the existing repertoire: JN.1 infection showed broader repertoire remodeling across V/J usage, IGHV-IGHJ pairing architecture, and antibody class composition, whereas BF.7 induced a more restricted pattern. This interpretation is consistent with longitudinal studies showing that SARS-CoV-2 infection and vaccination can drive continued maturation of memory B-cell responses and improve antibody breadth over time rather than relying solely on newly generated responses (30). Our data extend this concept to late-Omicron infection by showing that antigenically distinct lineages differ in the extent to which they recalibrate circulating BCR repertoires.

Importantly, the remodeling observed here did not resemble wholesale reconstruction of the BCR landscape. Major CDR3 length profiles, terminal sequence features, and light-chain repertoire architecture were largely preserved, while selected changes were observed in V-gene usage, diversity trends, isotype composition, and IGHV-IGHJ pairing weights. This pattern is compatible with studies showing that SARS-CoV-2 infection can generate convergent or expanded B-cell clonotypes within a broader pre-existing repertoire background (31). The concordance among stronger late-Omicron neutralization, antigenic map reorganization, and broader repertoire remodeling after JN.1 infection therefore supports a model of deeper humoral updating. This updating may be promoted by greater antigenic distance and does not require wholesale replacement of the B-cell repertoire.

The lineage-associated pattern observed here is relevant to vaccine antigen selection. Current vaccine updates have increasingly moved toward JN.1-lineage antigens, with KP.2- and LP.8.1-related recommendations reflecting continued tracking of the same evolutionary trajectory (20, 32). Recent evaluation of BNT162b2 JN.1- and KP.2-adapted vaccines provides a related example, showing improved antigenic alignment and neutralizing responses against JN.1 sublineages compared with earlier XBB.1.5-adapted formulations (33). Our data support this forward-looking strategy. Compared with BF.7, JN.1 infection was associated with stronger boosting against late-Omicron variants and broader repertoire remodeling, suggesting that antigens closer to the current antigenic front may more effectively update pre-existing humoral immunity. However, the retained resistance of BQ.1.1, XBB-derived variants, and KP.2 also argues against relying solely on matched-strain titers. Vaccine evaluation should include representative variants from BA.5-related, XBB-derived, and BA.2.86/JN.1-derived branches, especially branch-leading escape variants. This pattern supports vaccine strategies that prioritize breadth across antigenic space rather than only maximizing responses to the currently dominant lineage.

These observations also inform monoclonal antibody discovery and benchmarking. The clinical utility of SARS-CoV-2 monoclonal antibodies has repeatedly been limited by rapid variant escape, and even newer antibodies require continual reassessment against emerging JN.1 descendants (34). The persistent resistance of BQ.1.1 and KP.2 in our plasma neutralization analyses highlights the need to include branch-leading escape variants in antibody screening panels. In other words, activity against a parental or lineage-representative virus may not predict activity against the next antigenic step. Conversely, the broader neutralization and repertoire remodeling observed after JN.1 infection suggest that post-JN.1 convalescent repertoires may provide useful starting material for identifying antibodies with improved contemporary breadth. This rationale is consistent with studies showing that Omicron breakthrough infection can provide a source of potent pan-variant neutralizing antibodies (35). Although this study did not isolate monoclonal antibodies, our findings support a strategy in which exposure-shaped repertoires are mined for cross-lineage antibody candidates and evaluated against representative and prospective escape variants to define forward escape liability (36).

A strength of this study is its paired early- and convalescent-phase design, which allowed within-individual immune changes to be assessed rather than relying solely on cross-sectional comparisons. By analyzing convalescent-to-early changes in neutralization together with paired changes in repertoire diversity, V- and J-gene usage, isotype composition, and IGHV-IGHJ pairing weights, this design reduced the influence of baseline inter-individual variation. This paired framework was particularly informative in revealing greater neutralization gains and broader repertoire remodeling after JN.1 infection than after BF.7 infection.

Although Omicron breakthrough infection is known to broaden neutralization through recall of pre-existing memory B cells rather than primary-like priming (4, 9, 10), prior longitudinal studies of BA.5/BF.7/XBB breakthrough infection or reinfection have mainly defined humoral and cellular immune kinetics over time (37). Whether antigenically separated late-Omicron exposures differentially reshape humoral antibody recognition and BCR repertoires has remained unclear. To address this gap, we integrated neutralization profiling, antigenic cartography, and BCR repertoire analysis, revealing that JN.1 infection was associated with stronger late-Omicron response, more directional antigenic reorganization, and broader repertoire remodeling than BF.7 infection. This regional comparison links local lineage circulation to the direction of humoral updating and highlights infecting-variant antigenicity as a key axis shaping recall immunity.

Several limitations should be considered. The modest cohort size limited statistical power, particularly for adjusted and subgroup analyses. We therefore interpret the findings as lineage-associated immune trajectories rather than definitive population-level effect estimates. Multiple testing further limited inference, especially for BCR repertoire features, which were interpreted as exploratory concordance rather than standalone statistical evidence. The two cohorts were also not fully age-matched, with older participants in the JN.1 group than in the BF.7 group. Although other baseline features were broadly comparable and exploratory correlation analyses did not show clear associations between age and selected neutralization or IGH repertoire measures, residual confounding from age or unmeasured immune history cannot be excluded. Prior exposure history also required careful interpretation. Three BF.7 participants were unvaccinated but reported prior clinically diagnosed COVID-19 without virological confirmation. We therefore retained them under the study definition of breakthrough infection, while acknowledging uncertainty in prior infection documentation. The sampling window was limited to early convalescence, precluding assessment of durability, and neutralization was measured using pseudoviruses rather than authentic viruses. The study also cannot determine whether the observed serological differences translate directly into differences in clinical protection against future infection or severe disease. In addition, bulk BCR sequencing cannot resolve antigen-specific B-cell subsets, paired heavy-light chain relationships, or the clonotypes directly responsible for plasma neutralization. Therefore, the repertoire data should be interpreted as population-level evidence of remodeling rather than direct proof of clonal mechanism. Finally, although BF.7 and JN.1 co-detection in Fujian provided an informative natural comparison, the study was not designed to define the transmission mechanisms underlying this local pattern. Despite these constraints, the concordance across cross-variant neutralization, lineage-stratified resistance, antigenic cartography, and BCR repertoire analyses support the conclusion that BF.7 and JN.1 infections leave distinct humoral imprints. Later antigenically shifted lineages such as JN.1 may provide informative templates for future vaccine updating, immune-escape surveillance, and repertoire-guided antibody evaluation.

## Data Availability

The raw sequence data reported in this paper have been deposited in the Genome Sequence Archive (38) in National Genomics Data Center (39), China National Center for Bioinformation / Beijing Institute of Genomics, Chinese Academy of Sciences (GSA-Human: HRA020225) that are publicly accessible at https://ngdc.cncb.ac.cn/gsa-human.

## Supplementary material

Supplementary Figure 1 Individual-level neutralization profiles in the BF.7 breakthrough infection cohort. **(A)** Neutralization curves of early-infection plasma from individual BF.7-infected participants against the indicated pseudoviruses. **(B)** Neutralization curves of matched convalescent plasma from the same participants. Dotted lines indicate 50% neutralization.

Supplementary Figure 2 Individual-level neutralization profiles in the JN.1 breakthrough infection cohort. **(A)** Neutralization curves of early-infection plasma from individual JN.1-infected participants against the indicated pseudoviruses. **(B)** Neutralization curves of matched convalescent plasma from the same participants. Dotted lines indicate 50% neutralization.

Supplementary Figure 3 Light-chain repertoire features after BF.7 and JN.1 breakthrough infection. **(A)** Productive IGK and IGL read counts. **(B)** IGK and IGL clonotype counts. **(C)** Shannon diversity of IGK and IGL repertoires. **(D)** IGKV-IGKJ and IGLV-IGLJ pairing shown by chord diagrams.

Supplementary Figure 4 Additional isotype and light-chain CDR3 features after BF.7 and JN.1 breakthrough infection. **(A)** Paired comparisons of IgM, IgD, IgG1, IgG2, IgG3, and IgG4 proportions. **(B)** IGK and IGL CDR3 length distributions. **(C)** Density profiles of IGK and IGL CDR3 length.

Supplementary Figure 5 IGH CDR3 sequence logos across shorter and intermediate length bins. **(A-J)** Length-stratified productive IGH CDR3 amino-acid sequences are shown for BF.7 early, BF.7 convalescent, JN.1 early, and JN.1 convalescent samples. Letters are scaled by positional amino-acid frequency. n denotes the number of sequences in each logo.

Supplementary Figure 6 IGH CDR3 sequence logos across longer length bins. **(A-F)** Length-stratified productive IGH CDR3 amino-acid sequences are shown as in **Supplementary Figures 5**. Letters are scaled by positional amino-acid frequency. n denotes the number of sequences in each logo.

Supplementary Figure 7 Light-chain CDR3 sequence logos after BF.7 and JN.1 breakthrough infection. **(A-D)** Length-stratified IGK CDR3 sequence logos. **(E-J)** Length-stratified IGL CDR3 sequence logos. Letters are scaled by positional amino-acid frequency. n denotes the number of sequences in each logo

Supplementary Table 1 Age correlations with neutralization and IGH repertoire. Measures Abbreviations: ns, not significant (P > 0.05); ID_50_, 50% inhibitory dilution; IGH, immunoglobulin heavy chain; Δ, change from early to convalescent phase (Convalescent-Early).

Supplementary Table 2 FDR-adjusted q-values for pairwise ID50 comparisons in the BF.7 convalescent cohort. Note: Benjamini-Hochberg FDR correction was performed with q = 0.05 and applied independently to each (**Figure 3A**). N indicates the number of tests in the correction group; i indicates the rank of the raw P value within that group; ns indicates not significant. Comparisons with FDR q-value < 0.05 are considered significant.

Supplementary Table 3 FDR-adjusted q-values for pairwise ID50 comparisons in the JN.1 convalescent cohort. Note: Benjamini-Hochberg FDR correction was performed with q = 0.05 and applied independently to each **Figure 3B** panel. N indicates the number of tests in the correction group; i indicates the rank of the raw P value within that group; ns indicates not significant. Comparisons with FDR q-value < 0.05 are considered significant.

Supplementary Table 4 FDR-adjusted q-values for BCR clonal diversity comparisons. Note: For BCR clonal diversity analyses, FDR correction was performed using the Benjamini-Hochberg procedure with q = 0.05 and applied independently to each comparison type within each parameter category. N indicates the number of tests in the correction group; i indicates the rank of the raw P value; ns indicates not significant. Comparisons with FDR q-value < 0.05 are considered significant.

Supplementary Table 5 FDR-adjusted q-values for BCR isotype-switching comparisons. Note: For BCR isotype-switching analyses, FDR correction was performed using the Benjamini-Hochberg procedure with q = 0.05 and applied independently to each comparison type within each parameter category. N indicates the number of tests in the correction group; i indicates the rank of the raw P value; ns indicates not significant. Comparisons with FDR q-value < 0.05 are considered significant.

Supplementary Table 6 FDR-adjusted q-values for BCR CDR3 length comparisons. Note: For BCR CDR3 length analyses, FDR correction was performed using the Benjamini-Hochberg procedure with q = 0.05 and applied independently to each comparison type within each parameter category. N indicates the number of tests in the correction group; i indicates the rank of the raw P value; ns indicates not significant. Comparisons with FDR q-value < 0.05 are considered significant.
