# Supplementary material for: Comparative analysis of immune escape and BCR repertoire remodeling after Omicron BF.7 and JN.1 infections
Source: Front Immunol. 2026 Aug 25;17:1906350. doi: 10.3389/fimmu.2026.1906350 (PMC13550182; doi:10.3389/fimmu.2026.1906350)
Supplement: Supplementary file 1 [file Table1.docx]

**Supplementary Tables S1. Age correlations with neutralization and IGH repertoire measures**

| **Domain** | **Parameter** | **n** | **Spearman's rho** | **P value** | **Significance** |
| --- | --- | --- | --- | --- | --- |
| Neutralization | Age vs. JN.1 Fold change | 20 | 0.192 | 0.418 | ns |
|  | Age vs. JN.1 ID_50_ (Convalescent) | 20 | -0.240 | 0.308 | ns |
|  | Age vs. BF.7 Fold change | 20 | -0.165 | 0.487 | ns |
|  | Age vs. BF.7 ID_50_ (Convalescent) | 20 | -0.026 | 0.912 | ns |
| BCR repertoire | Age vs. IGH Clonotypes (Convalescent) | 18 | -0.059 | 0.816 | ns |
|  | Age vs. IGH Shannon (Convalescent) | 18 | 0.017 | 0.948 | ns |
|  | Age vs. Δ Clonotypes (Convalescent-Early) | 18 | -0.324 | 0.190 | ns |
|  | Age vs. Δ Shannon diversity (Convalescent-Early) | 18 | 0.013 | 0.958 | ns |

**Abbreviations:** ns, not significant (P > 0.05); ID_50_, 50% inhibitory dilution; IGH, immunoglobulin heavy chain; Δ, change from early to convalescent phase (Convalescent-Early).
