# Supplementary material for: Comparative analysis of immune escape and BCR repertoire remodeling after Omicron BF.7 and JN.1 infections
Source: Front Immunol. 2026 Aug 25;17:1906350. doi: 10.3389/fimmu.2026.1906350 (PMC13550182; doi:10.3389/fimmu.2026.1906350)
Supplement: Supplementary file 2 [file Table2.docx]

**Supplementary Table S2. FDR-adjusted q-values for pairwise ID50 comparisons in the BF.7 convalescent cohort**

| **Panel** | **Comparison** | **N** | **Rank (i)** | **Raw P** | **FDR q-value** | **Significant (q < 0.05)** |
| --- | --- | --- | --- | --- | --- | --- |
| Upper | BF.7 vs BQ.1.1 | 6 | 1 | 0.002 | 0.012 | Yes |
|  | BA.4/5 vs BQ.1.1 | 6 | 2 | 0.002 | 0.006 | Yes |
|  | BA.2 vs BQ.1.1 | 6 | 3 | 0.002 | 0.004 | Yes |
|  | BA.2 vs BF.7 | 6 | 4 | 0.037 | 0.0555 | No |
|  | BA.2 vs BA.4/5 | 6 | 5 | ns | >0.05 | No |
|  | BA.4/5 vs BF.7 | 6 | 6 | ns | >0.05 | No |
| Middle | XBB.1.16 vs EG.5.1 | 10 | 1 | 0.037 | 0.37 | No |
|  | Remaining 9 comparisons | 10 | 2–10 | ns | >0.05 | No |
| Lower | JN.1 vs KP.2 | 3 | 1 | 0.002 | 0.006 | Yes |
|  | BA.2.86 vs KP.2 | 3 | 2 | 0.0039 | 0.00585 | Yes |
|  | BA.2.86 vs JN.1 | 3 | 3 | ns | >0.05 | No |

Note: Benjamini-Hochberg FDR correction was performed with q = 0.05 and applied independently to each Figure 3A panel. N indicates the number of tests in the correction group; i indicates the rank of the raw P value within that group; ns indicates not significant. Comparisons with FDR q-value < 0.05 are considered significant.
