# Supplementary material for: Comparative analysis of immune escape and BCR repertoire remodeling after Omicron BF.7 and JN.1 infections
Source: Front Immunol. 2026 Aug 25;17:1906350. doi: 10.3389/fimmu.2026.1906350 (PMC13550182; doi:10.3389/fimmu.2026.1906350)
Supplement: Supplementary file 4 [file Table4.docx]

**Supplementary Table S4. FDR-adjusted q-values for BCR clonal diversity comparisons**

| **Parameter** | **Comparison Mode** | **Comparison** | **N** | **Rank(i)** | **Raw P** | **q-value** | **Significant** |
| --- | --- | --- | --- | --- | --- | --- | --- |
| IGK Reads | independent | BF.7 vs JN.1 Early | 2 | 1 | 0.0001 | 0.0002 | Yes |
| IGK Reads | independent | BF.7 vs JN.1 Convalescent | 2 | 2 | 0.0188 | 0.0188 | Yes |
| IGL Reads | independent | BF.7 vs JN.1 Early | 2 | 1 | 0.0017 | 0.0034 | Yes |
| IGH Reads | independent | BF.7 vs JN.1 Early | 2 | 1 | 0.0346 | 0.0692 | No |
| Other Reads | independent | Other 2 comparisons | 2 | 1-2 | ns | >0.05 | No |
| All Clonotypes | independent | All 6 comparisons | 2 | 1-2 | ns | >0.05 | No |
| All Shannon | independent | All 6 comparisons | 2 | 1-2 | ns | >0.05 | No |
| IGH Clonotypes | paired | JN.1 Early vs Convalescent | 2 | 1 | 0.0352 | 0.0704 | No |
| IGK Clonotypes | paired | JN.1 Early vs Convalescent | 2 | 1 | 0.0286 | 0.0572 | No |
| IGL Clonotypes | paired | JN.1 Early vs Convalescent | 2 | 1 | 0.0253 | 0.0506 | No |
| Other Clonotypes | paired | Other 3 comparisons | 2 | 1-2 | ns | >0.05 | No |
| All Reads | paired | All 6 comparisons | 2 | 1-2 | ns | >0.05 | No |
| IGH Shannon | paired | JN.1 Early vs Convalescent | 2 | 1 | 0.034 | 0.068 | No |
| Other Shannon | paired | Other 5 comparisons | 2 | 1-2 | ns | >0.05 | No |

Note: For BCR clonal diversity analyses, FDR correction was performed using the Benjamini-Hochberg procedure with q = 0.05 and applied independently to each comparison type within each parameter category. N indicates the number of tests in the correction group; i indicates the rank of the raw P value; ns indicates not significant. Comparisons with FDR q-value < 0.05 are considered significant.
