# Supplementary material for: Comparative analysis of immune escape and BCR repertoire remodeling after Omicron BF.7 and JN.1 infections
Source: Front Immunol. 2026 Aug 25;17:1906350. doi: 10.3389/fimmu.2026.1906350 (PMC13550182; doi:10.3389/fimmu.2026.1906350)
Supplement: Supplementary file 6 [file Table6.docx]

**Supplementary Table S6. FDR-adjusted q-values for BCR CDR3 length comparisons**

| **Parameter** | **Comparison Mode** | **Comparison** | **N** | **Rank (i)** | **Raw P** | **FDR q-value** | **Significant** |
| --- | --- | --- | --- | --- | --- | --- | --- |
| IGH Median Length | independent | BF.7 vs JN.1 Early | 2 | 1 | 0.0459 | 0.0918 | No |
| Other Median Length | independent | Other 11 comparisons | 2 | 1-2 | ns | >0.05 | No |
| All Median Length | paired | All 12 comparisons | 2 | 1-2 | ns | >0.05 | No |

Note: For BCR CDR3 length analyses, FDR correction was performed using the Benjamini-Hochberg procedure with q = 0.05 and applied independently to each comparison type within each parameter category. N indicates the number of tests in the correction group; i indicates the rank of the raw P value; ns indicates not significant. Comparisons with FDR q-value < 0.05 are considered significant.
